# Apparent Survival Rates of Forest Birds in Eastern Ecuador Revisited: Improvement in Precision but No Change in Estimates

**DOI:** 10.1371/journal.pone.0081028

**Published:** 2013-12-02

**Authors:** John G. Blake, Bette A. Loiselle

**Affiliations:** 1 Department of Wildlife Ecology and Conservation, University of Florida, Gainesville, Florida, United States of America; 2 Center for Latin American Studies, University of Florida, Gainesville, Florida, United States of America; University of Missouri-Columbia, United States of America

## Abstract

Knowledge of survival rates of Neotropical landbirds remains limited, with estimates of apparent survival available from relatively few sites and species. Previously, capture-mark-recapture models were used to estimate apparent survival of 31 species (30 passerines, 1 Trochilidae) from eastern Ecuador based on data collected from 2001 to 2006. Here, estimates are updated with data from 2001-2012 to determine how additional years of data affect estimates; estimates for six additional species are provided. Models assuming constant survival had highest support for 19 of 31 species when based on 12 years of data compared to 27 when based on six; models incorporating effects of transients had the highest support for 12 of 31 species compared to four when based on 12 and six years, respectively. Average apparent survival based on the most highly-supported model (based on model averaging, when appropriate) was 0.59 (± 0.02 SE) across 30 species of passerines when based on 12 years and 0.57 (± 0.02) when based on six. Standard errors of survival estimates based on 12 years were approximately half those based on six years. Of 31 species in both data sets, estimates of apparent survival were somewhat lower for 13, somewhat higher for 17, and remained unchanged for one; confidence intervals for estimates based on six and 12 years of data overlapped for all species. Results indicate that estimates of apparent survival are comparable but more precise when based on longer-term data sets; standard error of the estimates was negatively correlated with numbers of captures (*r_s_* = −0.72) and recaptures (*r_s_* = −0.93, *P*<0.001 in both cases). Thus, reasonable estimates of apparent survival may be obtained with relatively few years of data if sample sizes are sufficient.

## Introduction

Accurate estimates of survival rates are necessary for advancing our understanding of life-history strategies of tropical birds and how those strategies might differ from comparable temperate species [1] or among regions of the tropics. Early estimates of high survival rates in some tropical birds (e.g., male manakins, Pipridae, on leks [2], [3]) have given way to an understanding that survival rates vary geographically and among species, with many estimates considerably lower than those early ones [4]–[8]. Yet, estimates of survival rates of tropical birds still are limited and often based on relatively few data so that standard errors of the estimates are often high [9].

Earlier we published estimates of survival rates for 31 species of birds found in lowland Ecuador, based on 6 years of data [8]. Our estimate of average survival fell between estimates from sites in Central America and two sites in South America. Here, we update that study with a reexamination of estimates now based on 12 years of data from the same sites. The basic question we ask is how an additional six years of data changes our perspectives on estimates of survival rates. Understanding how and if survival estimates are affected by the length of the study [10] is important in order to reach conclusions regarding evolution of life history characteristics [11]. From a practical standpoint, if estimates do not change significantly with a doubling of sample effort, then results from shorter-term studies may be sufficient to provide reasonable estimates. Previous studies that have used capture-recapture analyses to estimate survival rates of tropical birds have been based on from 4 to 21 years of data (see [8] for a review). Although we use the term survival for convenience, it is important to note that we actually estimate ‘apparent survival’ given that live-encounter data (capture-mark-recapture or capture-mark-resight) can fail to distinguish true survival (mortality) from permanent emigration from the study area [12]. Estimates are, as a consequence, a product of true survival and site fidelity and will underestimate true survival by some unknown amount [13].

## Methods

### Ethics Statement

This study was reviewed and approved by the Institutional Animal Care and Use Committee of the University of Missouri - St. Louis and by the Institute of Food and Agricultural Science Research Administration Committee for Non-Regulatory Animal Research (018-10WEC), University of Florida. Research methods follow the Guidelines to the Use of Wild Birds in Research 2nd edition (Ornithological Council). Research at Tiputini Biodiversity Station was conducted in accordance with research permit number 13-IC-FAU-DFN (and renewals), Ministerio del Ambiente, Distrito Forestal Napo, Tena, Ecuador.

### Study Site

Research was conducted at Tiputini Biodiversity Station (TBS), Orellana Province, Ecuador (*ca* 0°37′ S, 76°10′ W, 190–270 m asl). TBS is located on a tract of undisturbed lowland rain forest within Yasuní Biosphere Reserve, one of the most diverse regions of the world [14]. The station and nearby areas are dominated by *terra firme* forest; *várzea* forest, palm swamps, and various successional habitats also are present. Mean annual precipitation at Yasuní Research Station, approximately 30 km WSW of TBS, is about 3100 mm.

### Bird Sampling

We established two 100-ha plots (*ca* 1 × 1 km each) in *terra firme* forest during 2001. Both plots were gridded (100×200-m grid lines) and marked with 1.5-m PVC tubes. The Harpia plot ranges from 201 to 233 m elevation and is characterized by more dissected upland forests. The Puma plot is flatter overall although the elevation range is similar (209 to 235 m). Both areas experience partial inundation when the Tiputini River rises; Puma has more areas that fill with persistent standing water during the rainy season.

Birds were captured with mist nets (12×2.6 m, 36-mm mesh) set at ground level. Nets were arranged in a series of 8 sets of 12 nets on each plot; each set of 12 nets formed a rectangle (100 × 200 m) with nets set *ca* 50 m apart. Nets on a given plot were ∼920 m apart at the farthest point whereas nets on the two plots were *ca* 1.7 km apart at their closest point. Each set of nets was run for one day (∼0600 to ∼1230 h) in January (peak of breeding for many species) and March (late breeding season for many species), starting in March 2001. All captured birds were identified, sexed and aged (when possible), and banded with uniquely numbered aluminum leg bands. Taxonomy follows Remsen et al. [15].

### Analyses

We estimated apparent survival for species represented by at least 20 recaptures (18 for one species) and 40 individuals; previously we used 20 individuals as a minimum [8]. We created a capture history for each individual based on captures and recaptures during each of the sample periods (23 capture periods, 22 capture intervals) and used capture-mark-recapture analyses (based on Cormack-Jolly-Seber models) for open populations to estimate annual apparent survival and recapture rates [10]. All analyses were run using Program MARK [16], [17] with capture intervals set to 0.17 or 0.83 years (2 or 10 months).

Following our earlier paper [8], we evaluated a series of six *a priori* models that differed in assumptions regarding constancy of apparent survival and recapture rates (Table 1). Different models assumed that: (1) apparent survival (*φ*) varied across sampling periods but was the same for all individuals (i.e., fully time-specific); (2) survival remained constant over time and the same for all individuals; or (3) survival in the first interval after initial capture (*φ*_1_) differed from survival during the second (*φ*_2_) and subsequent capture intervals (Time-Since-Marking models) [17]. TSM models account for possible effects of birds that simply move through the study area, with little likelihood of recapture (i.e. transients); inclusion of such birds may decrease the overall estimate of apparent survival [5] [7], [18], [19]. Recapture rates were assumed to either vary or remain constant over time (Table 1). We tested the most general model under consideration for goodness-of-fit (GOF) using Program U-CARE V2.2 [17], [20]. U-CARE includes a test for transience that can be used to investigate the effect of individuals (transients) moving through the area (similar to the TSM model); to determine the effect of transients, we ran the GOF tests with and without the first capture. U-CARE also includes a test for ‘trap-shyness’ which can be used to evaluate the suggestion that individual birds may learn locations of mist nets and, therefore, have a reduced chance of being recaptured over time [6].

**Table 1:** Descriptions and notations for Cormack-Jolly-Seber (CJS) models used to estimate apparent survival and recapture.

Surv.	Rec.	Par.	Model description
*φ*(.)	*p*(.)	2	constant survival; constant recapture
*φ*(2./.)	*p*(.)	3	Time-Since-Marking model (TSM); two classes for survival (first and subsequent intervals after marking) with survival constant for each class; constant recapture
*φ*(.)	*p*(t)	23	constant survival; time-dependent recapture
*φ*(2./.)	*p*(t)	24	TSM – survival of both classes constant; time-dependent recapture
*φ*(t)	*p*(.)	23	time-dependent survival; constant recapture
*φ*(t)	*p*(t)	44	standard CJS model, time-dependent survival and recapture

Surv.  =  apparent survival, Rec.  =  recapture, Par.  =  number of parameters.

Model selection was evaluated with Akaike = s Information Criteria, with adjustment for small sample sizes (AIC_c_) and overdispersion of the data (QAIC_c_), when necessary [21]. Calculation of overdispersion was based on GOF chi-square from U-CARE divided by degrees of freedom [17]. Models with an AIC_c_ difference within ∼ 2 from the best model were considered to have substantial, and relatively equal, support (following [21]) except when models were within 2 units of the minimum model and incorporated one extra parameter (see [21:131]). Means are reported with ± SE. When there was more than one competitive model, we used model-averaged results in subsequent comparisons.

We used parametric tests when data fit appropriate assumptions and nonparametric tests when data did not fit assumptions of parametric tests (even after transformations) but did meet assumptions of nonparametric tests. We used *t*-tests to compare number of captures and recaptures for species best represented by TSM versus those best represented by constant-survival models. We used correlation analyses (Spearman’s *r*) to examine relationships between standard errors of survival estimates and numbers of captures and recaptures and used paired *t*-tests to compare standard errors based on six versus 12 years of data. Finally, we used chi-square tests to compare the numbers of species best represented by TSM models in 2006 versus 2012. Data will be deposited in Dryad Digital Repository.

## Results

### 2012 Results

We recorded ∼12,455 captures (excluding birds recaptured within a sample period and excluding birds only captured during the final sample) of 177 species from March 2001 through March 2012 but most species were represented by too few captures to model apparent survival. Here, we present estimates for 37 species (Table 2); these include six species not analyzed previously. Goodness-of-fit tests indicated that the most general model adequately represented the data for all species. Evidence of minor overdispersion was detected only for *Glyphorynchus spirurus* (c-hat  =  1.45); QAIC_c_ values were used to rank models for this species. GOF tests (one-tailed test of significance) indicated that transients were important for 10 species (*Phaethornis malaris, Automolus infuscatus, Glyphorynchus spirurus, Philydor erythrocercum, Gymnopithys leucaspis, Myrmoborus myotherinus, Epinocrophyla fjeldsaai, Pithys albifrons, Lepidothrix coronata, Pipra filicauda*). There was no indication (*P* > 0.20, based on U-CARE tests) that net-avoidance was a problem for any species.

**Table 2:** Apparent survival rate (*φ*) estimates (and standard errors) are based on data collected on two 100-ha plots in Ecuador, 2001–2012.

		2001 – 2012 data							2001 – 2006 data			
Species	Model	I/R^a^	ΔAIC_c_ b	*w_i_* c	*φ_1_*	SE	*φ_2_*	SE	ΔAIC_c_	*w_i_*	*φ_2_*	SE
**Nonpasserines**												
**Trochilidae**												
*Phaethornis malaris*	*φ*(2./.)*p*(.)	265/100	0.0	1.0	0.08	0.03	0.42	0.05	0.0	0.99	0.53	0.08
**Bucconidae**												
*Malacoptila fusca*	*φ*(.)*p*(.)	57/22	0.0	0.67	0.61	0.08	0.61	0.08				
**Passerines**												
**Furnariidae**												
*Automolus infuscatus*	*φ*(2./.)*p*(.)	190/158	0.0	0.74	0.34	0.07	0.54	0.04				
	*φ*(.)*p*(.)								0.0	0.68	0.48	0.05
*Glyphorynchus spirurus*	*φ*(2./.)*p*(.)	892/916	0.0	0.99	0.41	0.03	0.62	0.02	0.0	0.90	0.59	0.03
*Hyloctistes subulatus*	*φ*(.)*p*(.)	72/35	0.0	0.71	0.57	0.06	0.57	0.06	0.0	0.64	0.59	0.14
*Philydor erythrocercum*	*φ*(.)*p*(.)	87/36	0.0	0.71	0.53	0.06	0.53	0.06	0.0	0.75	0.63	0.10
*Sclerurus caudacutus*	*φ*(.)*p*(.)	62/57	0.0	0.66	0.59	0.05	0.59	0.05	0.0	0.70	0.66	0.08
*Xenops minutus*	*φ*(2./.)*p*(.)	85/60	0.0	0.54	0.43	0.14	0.71	0.05				
	*φ*(.)*p*(.)		0.33	0.46	0.68	0.04	0.68	0.04	0.0	0.51	0.52	0.12
*Xiphorhynchus ocellatus*	*φ*(.)*p*(.)	75/62	0.0	0.59	0.70	0.04	0.70	0.04	0.0	0.61	0.72	0.09
**Thamnophilidae**												
*Hylophylax naevius*	*φ*(.)*p*(.)	244/101	0.0	0.59	0.69	0.03	0.69	0.03	0.0	0.71	0.73	0.07
*Epinecrophylla fjeldsaai*	*φ*(.)*p*(.)	94/42	0.0	0.56	0.55	0.06	0.55	0.06	0.0	0.75	0.53	0.14
*Gymnopithys leucaspis*	*φ*(2./.)*p*(.)	124/91	0.0	0.55	0.36	0.09	0.55	0.05				
	*φ*(.)*p*(.)		0.43	0.45	0.51	0.04	0.51	0.04	0.0	0.72	0.56	0.06
*Myrmeciza fortis*	*φ*(.)*p*(.)	71/18	0.0	0.75	0.65	0.08	0.65	0.08				
*Myrmoborus myotherinus*	*φ*(.)*p*(.)	195/71	0.0	0.72	0.60	0.04	0.60	0.04	0.0	0.48	0.59	0.08
*Myrmotherula axillaris*	*φ*(2./.)*p*(.)	121/28	0.0	0.85	0.15	0.10	0.69	0.08				
*Myrmotherula hauxwelli*	*φ*(2./.)*p*(.)	171/90	0.0	0.53	0.90	0.19	0.62	0.04				
	*φ*(.)*p*(.)		0.23	0.47	0.65	0.04	0.65	0.04	0.0	0.69	0.64	0.09
*Myrmotherula longipennis*	*φ*(.)*p*(.)	103/44	0.0	0.71	0.51	0.06	0.51	0.06	0.0	0.68	0.47	0.10
*Pithys albifrons*	*φ*(2./.)*p*(.)	278/214	0.0	0.94	0.24	0.05	0.47	0.04	0.69	0.22	0.43	0.06
	*φ*(2./.)*p*(t)								0.0	0.31	0.42	0.06
*Thamnomanes ardesiacus*	*φ*(.)*p*(.)	241/91	0.0	0.56	0.60	0.04	0.60	0.04	0.51	0.43	0.60	0.07
	*φ*(2./.)*p*(.)								0.0	0.55	0.67	0.08
*Thamnomanes caesius*	*φ*(.)*p*(.)	235/43	0.0	0.69	0.46	0.06	0.46	0.06	0.0	0.72	0.57	0.13
*Willisornis poecilinotus*	*φ*(.)*p*(.)	309/224	0.0	0.73	0.56	0.03	0.56	0.03	0.0	0.66	0.55	0.04
**Conopophagidae**												
*Conopophaga peruviana*	*φ*(.)*p*(.)	104/28	0.0	0.61	0.57	0.07	0.57	0.07				
**Formicariidae**												
*Formicarius colma*	*φ*(.)*p*(.)	65/38	0.0	0.72	0.44	0.06	0.44	0.06	0.0	0.76	0.55	0.09
**Tyrannidae**												
*Corythopis torquatus*	*φ*(.)*p*(.)	61/20	0	0.69	0.64	0.08	0.64	0.08				
*Mionectes oleagineus*	*φ*(.)*p*(.)	169/58	0.0	0.65	0.45	0.05	0.45	0.05	1.62	0.23	0.32	0.09
	*φ*(.)*p*(t)								0.0	0.52	0.32	0.09
*Myiobius barbatus*	*φ*(2./.)*p*(.)	74/41	0.0	0.69	0.34	0.13	0.71	0.06				
	*φ*(.)*p*(.)		1.56	0.31	0.65	0.05	0.65	0.05	0.0	0.68	0.68	0.09
*Platyrinchus coronatus*	*φ*(.)*p*(.)	78/62	0.0	0.71	0.61	0.05	0.61	0.05	0.0	0.74	0.57	0.10
**Pipridae**												
*Chiroxiphia pareola*	*φ*(2./.)*p*(.)	90/63	0.0	0.84	0.37	0.12	0.76	0.05				
	*φ*(.)*p*(.)								0.0	0.74	0.55	0.09
*Lepidothrix coronata*	*φ*(2./.)*p*(.)	445/222	0.0	0.65	0.44	0.07	0.61	0.03				
	*φ*(.)*p*(.)		1.27	0.35	0.58	0.02	0.58	0.02	0.0	0.70	0.58	0.04
*Pipra filicauda*	*φ*(2./.)*p*(.)	226/153	0.0	0.64	0.45	0.09	0.69	0.03				
	*φ*(.)*p*(.)		1.93	0.24	0.66	0.03	0.66	0.03	0.0^d^	0.69	0.60	0.06
*Dixiphia pipra*	*φ*(2./.)*p*(.)	173/76	0.0	0.52	0.41	0.11	0.63	0.05				
	*φ*(.)*p*(.)		0.18	0.48	0.60	0.04	0.60	0.04	0.0	0.73	0.52	0.09
**Vireonidae**												
*Hylophilus ochraceiceps*	*φ*(.)*p*(.)	53/25	0.0	0.75	0.68	0.07	0.68	0.07				
**Troglodytidae**												
*Henicorhina leucosticta*	*φ*(.)*p*(.)	66/31	0.0	0.70	0.80	0.06	0.80	0.06	0.0	0.73	0.80	0.15
*Microcerculus marginatus*	*φ*(.)*p*(.)	47/39	0.0	0.56	0.51	0.07	0.51	0.07	0.0	0.72	0.50	0.11
**Turdidae**												
*Turdus albicollis*	*φ*(.)*p*(.)	97/72	0.0	0.69	0.66	0.04	0.66	0.04	0.0	0.69	0.57	0.08
**Cardinalidae**												
*Habia rubica*	*φ*(.)*p*(.)	52/29	0.0	0.74	0.64	0.07	0.64	0.07	0.0	0.76	0.65	0.12
*Cyanocompsa cyanoides*	*φ*(.)*p*(.)	59/38	0.0	0.56	0.43	0.06	0.43	0.06	0.0	0.71	0.50	0.12

Results are based on the first six years of data (2001–2006; see [8]) and for the full 12 years. Competitive models (ΔAIC_c_<2.0) are ordered by AICc rankings for the full 12-year results; corresponding results from the reduced data set of 2006 follow that ranking (estimates for some species were not calculated for the reduced data set). Estimates are shown for both the first (*φ_1_*) and subsequent capture periods [*φ_2_*, i.e., TSM models, e.g. *φ*(2./.)p(.)] for the full data set (2001–2012) but only *φ_2_* for the reduced set (2001–2006 data).

a I/R - number of individuals captured/number of recaptures (excluding individuals only captured during the final sample) over the 12-year period.

b ΔAIC_c_ - differences in AIC_c_.

c *w_i_* - relative strength (weight) of evidence for selected models.

d Model included *p*(t) rather than *p*(.).

Models with constant apparent survival had the most support (highest AIC_c_ weight) for 24 species (Table 2). TSM models had the most support for 13 species; models with constant survival were well-supported (i.e. AIC_c_ difference within ∼ 2) for seven of those. Excluding *Glyphorynchus spirurus* (with more than twice as many captures and recaptures as any other species), mean number of captures was higher for TSM-model species (187±30.6 vs 112±15.4; *t* = 2.76, df = 34, *P*<0.01) as was number of recaptures (108±18.6 vs 54±8.6; *t* = 3.37, df = 34, *P*<0.01; tests based on log-transformed data). Models including time-dependence were not well supported for any species.

Based on the most highly supported model, mean apparent survival during the first interval after capture (*φ*_1_) was lower across all 35 passerines (0.21±0.03) than during the second (*φ*_2_) and subsequent intervals (0.60±0.02). Estimates of apparent survival (*φ*_2_) from the most highly supported model varied from 0.43 to 0.80 (Table 2); estimates were > 0.7 for five species.

Standard errors for estimates of apparent survival ranged from 0.08 (four species) to 0.02 (*Glyphorynchus spirurus*) when based on the most highly supported model and were negatively correlated both with numbers of captures (*r_s_* = −0.72) and numbers of recaptures (*r_s_* = −0.93; *P*<0.001, both cases

### Comparisons With 2006 Results

**Figure 1:**
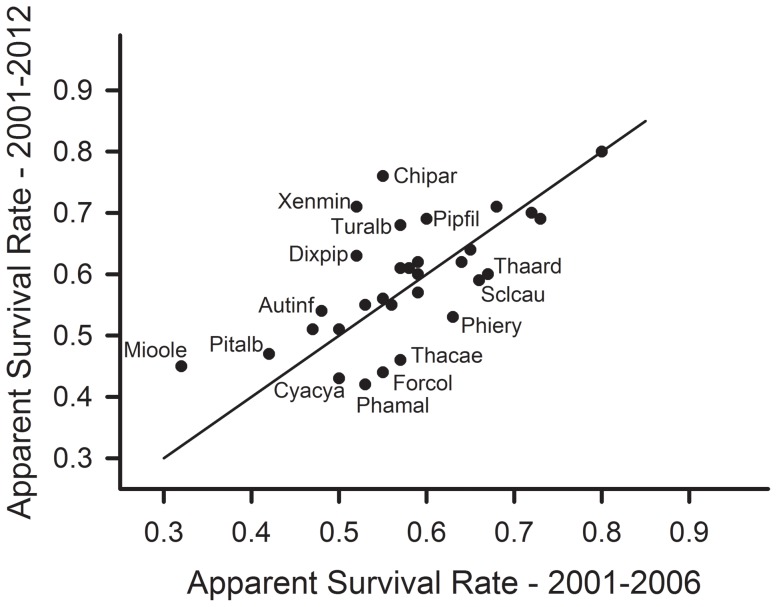
Estimates of apparent survival for 31 species based on 6 or 12 years of data. Estimates of apparent survival were calculated from capture and recapture data gathered at Tiputini Biodiversity Station, Ecuador. Estimates shown are for the most highly supported model. Straight line indicates equal estimates between the two sets of data. Species showing substantial differences between sets: Autinf – *Automolus infuscatus*; Chipar – *Chiroxiphia pareola*; Cyacya – *Cyanocompsa cyanoides*; Forcol – *Formicarius colma*; Mioole – *Mionectes oleagineus*; Phamal – *Phaethornis malaris*; Phiery – *Philydor erythrocercum*; Pipfil – *Pipra filicauda*; Dixpip – *Dixiphia pipra*; Pitalb - *Pithys albifrons*; Sclcau – *Sclerurus caudacutus*; Thaard - *Thamnomanes ardesiacus*; Thacae – *Thamnomanes caesius*; Turalb – *Turdus albicollis*; Xenmin – *Xenops minutus*.

In the following, most analyses are based on comparisons of data from 30 passerine species whose apparent survival rates were estimated both with the first 6 years of data (referred to as 2006) and all 12 years (2012). TSM models accounted for a greater proportion of most highly supported models during 2012 (11 of 30) than during 2006 (3 of 30) (χ^2^ = 6.0, df = 1, *P*<0.05). Based on the most highly supported model, mean apparent survival (*φ*_2_) across 30 species did not differ between 2006 (0.57±0.02) and 2012 (0.59±0.02). Standard errors of estimates of apparent survival (*φ*_2_ for the most highly supported model) were, however, lower in 2012 (0.05±0.002) than during 2006 (0.09±0.006) (*t* = 10.5, df = 29, *P*<0.001). Although estimates of apparent survival were lower in 2012 for 12 of 30 passerine species and higher for 17 (Table 2), confidence intervals overlapped for estimates based on 6 or 12 years of data. The absolute value of the mean decrease (0.06±0.011) did not differ from the mean increase (0.07±0.015). Seven species [including one nonpasserine, *Phaethornis malaris* (Trochilidae)] showed decreases of at least 10% (2012 relative to 2006); eight species increased by at least 10% (Fig. 1). Species that increased by at least 20% included *Mionectes oleagineus* (41%), *Chiroxiphia pareola* (38%), *Xenops minutus* (37%), and *Dixiphia pipra* (21%); only two species decreased by at least 20%, including *Phathornis malaris* (21%) and *Formicarius colma* (20%).

## Discussion

Twelve years of sampling approximately doubled (or more) the number of individuals and recaptures for the species included in both analyses ([8] and current study). Additional years of data also extended the known age of many individuals (e.g., during March 2013 we recaptured a male *Chiroxiphia pareola* that was first captured, as an adult in definitive plumage, in 2001). Nonetheless, estimates of apparent survival rates for birds at our site in western Amazonia were, with some exceptions, generally similar but more precise (lower standard errors) when analyses were based on 12 years of data rather than 6 [8]. Average survival rate (*φ*_2_) for 30 passerines showed no change (0.57 vs 0.59, based on most highly supported model). Only one species, *Henicorhina leucosticta*, had an estimate of at least 0.8 and showed no change between the two sets of data.

Estimates of apparent survival were lower for seven species and higher for 17 but the absolute values of the changes did not differ between the two groups. Estimates were at least 20% lower for two species (*Formicarius colma*, *Phaethornis malaris*) and higher by at least 20% for four species (*Xenops minutus, Chiroxiphia pareola, Dixiphia pipra, Mionectes oleagineus*). Based on the ecological characteristics of species showing the largest changes (up or down) in estimates of apparent survival (Fig. 1), there was no readily apparent pattern among those species; species with higher estimates included lek breeders, flock members, territorial species, and ground insectivores. Species with lower estimates included a lek breeder (*Phaethornis malaris*), flock members, ground insectivores, and territorial species. Given that confidence intervals of estimates based on 6 and 12 years of data overlapped to some extent for all species, even those that showed the greatest percentage change, it is possible that the apparent changes may be artifacts of sampling and may not represent real changes in apparent survival.

Some of the earliest estimates of high (>80%) survival rates in tropical birds were based on observations of adult male manakins (*Manacus manacus*) on leks [2], [3]. Most later estimates for manakins have been lower (∼0.50 – 0.77), whether based on captures or observations (reviewed in [8]; see also [22]). In this study, estimates of apparent survival were higher for all four species of manakins, although the increase for *Lepidothrix coronata* was smaller (7%) than for the other three species (*Pipra filicauda*, 11%; *Dixiphia pipra* 19%; *Chiroxiphia pareola*, 38%). Despite the changes, estimates for three species are still considerably lower than early estimates for *Manacus*. On the other hand, our current estimate for *Chiroxiphia pareola* (0.76) is similar to that obtained by [23] for *Chiroxiphia linearis* in Costa Rica (0.77, based on 10 years of resighting data). Yet, lek-breeding *per se* is not always associated with higher survival rates. *Phaethornis malaris* is a lek-breeding hummingbird but has an estimated survival rate of 0.42 (down from 0.53 based on our earlier study). The estimate for *Mionectes oleagineus*, a lek-breeding flycatcher, increased from our earlier study by 32% but was still low (0.45). Estimates of survival rates for *Mionectes* are generally low (0.35, Osa Peninsula, Costa Rica, [24]; 0.44, La Selva, Costa Rica, [8]; 0.53, Panama, [5]; but 0.62 in Trinidad, [18]).

Estimates of apparent survival rate may be low if transients are not accounted for in the analyses [5], [18]. In our previous analysis, models assuming constant survival (i.e., no effect of transients) had the highest support for 27 of 31 species [8]. In contrast, when analyses were based on 12 years of data, models assuming constant survival were more highly supported for 19 of the same set of 31 species; models with constant survival were most highly supported for 24 of all 37 species included in the present study. Longer-term studies that allow for inclusion of greater numbers of captures and recaptures may provide a better perspective on the apparent impact of transients. Of the six species added in this analysis, a constant model was most highly supported for five. These species were generally represented by relatively fewer captures and recaptures. In contrast, species with greater numbers of captures and recaptures were more likely to be best represented by TSM models, although estimates of survival often were not substantially different for the two models, which suggests that for many species, transients may not significantly affect estimates of apparent survival. Although time-dependent models were not supported in these analyses, it is important to remember that the relatively small samples sizes for many species, even after 12 years of data, make it harder for time-dependent models to be competitive.

Karr et al. [4] were the first to suggest that average survival rates of tropical birds were considerably lower than early studies indicated. That study was faulted (e.g., [18]) for not fully accounting for effects of transients. Yet, even after accounting for transients [5] average survival rate was still relatively low (*φ*_2_ = 0.58, based on 21 years of data) and similar to results from Costa Rica (*φ*_2_ = 0.56) based on 5 to 10 years of data ([7], [8], unpublished data). These rates are lower than those previously estimated for sites in South America (Peru: *φ*_2_ = 0.68, [6], 10 years; French Guiana: *φ*_2_ = 0.63, 4 years; [9]) and for islands (Puerto Rico: 0.68, 18 years, [25]; Trinidad: 0.65, 10 years, [18]). Our initial results from Ecuador [8] were based on 6 years of data and yielded an average apparent survival rate for 30 passerines of 0.57, more in line with results from Central America. The current estimate of average apparent survival of 0.59 for the most highly supported model is only slightly higher than previous estimates for Central American sites. Overall, our estimates of apparent survival were more precise (lower SE) when based on more years (and greater numbers of individuals and recaptures). Nonetheless, the range of estimates remained high (from ∼ 0.42 to 0.80), supporting previous conclusions [5] that survival rates vary substantially among tropical species and among tropical regions.

Recently, Ruiz-Gutiérrez et al. [11] recommended 10 to 30 years as an appropriate time frame for studies on population dynamics of tropical birds based partially on the assumptions that tropical species are long-lived but have low recapture probabilities. Yet, results of our study indicate that six years of sampling may be sufficient to estimate apparent survival for some species. Length of study needed to provide reliable estimates with low standard errors may depend on location of study and sampling design. At our site, capture rates have been higher (mean over 12 years of 53 captures per 100 mist-net-hours) than at most other sites in the tropics (see [26]) and, as a consequence, we are able to obtain reasonably large numbers of captures for many species. Further, our nets sample a relatively large area and recapture rates, partially as a consequence, are high (long-term average of ∼41%). Ruiz-Gutiérrez et al. [11] suggested that the low number of captures and low recapture rates in their study might have been related to the limited spatial extent of the net coverage (< 2 ha), combined with net avoidance brought on by frequent sampling and relatively few number of nets used. Larger areas will sample more territories and more complete territories, given that most tropical species have territories larger than 2 ha [27], [28] and, consequently, may increase the likelihood of recapturing individuals.

Longer time frames do have the benefit of providing a more precise estimate of apparent survival. Further, if sampling is relatively infrequent (e.g., 1 day/month, 2 months/year, as in our study) many individuals will not be recaptured in any given sample, even when present, but probability of recapture will increase with more years sampled. In conclusion, long-term studies provide important insights regarding variation in apparent survival rates both among species within a site and among geographic regions. Length of study needed to achieve good estimates of apparent survival also depends on the study design and rates at which birds are captured and recaptured.
